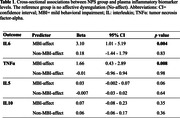# Association of later‐life emergent affective dysregulation with neuroinflammatory biomarkers in dementia‐free older adults

**DOI:** 10.1002/alz.095723

**Published:** 2025-01-09

**Authors:** Maryam Ghahremani, Zahinoor Ismail

**Affiliations:** ^1^ Hotchkiss Brain Institute, University of Calgary, Calgary, AB Canada

## Abstract

**Background:**

Neuroinflammation has emerged as a significant factor in the pathogenesis of dementia. Elevated levels of proinflammatory and anti‐inflammatory cytokines such as interleukin‐6 (IL6), interleukin‐10 (IL10), and tumor necrosis factor‐alpha (TNFα) have been observed in the cerebrospinal fluid and plasma of patients with Alzheimer’s disease, associating with cognitive decline and dementia. Here, we explored the cross‐sectional association between plasma inflammatory biomarkers and the affective dysregulation domain of Mild Behavioral Impairment (MBI), a validated neurobehavioral syndrome capturing later‐life emergent and persistent neuropsychiatric symptoms (NPS).

**Method:**

Data from dementia‐free older adults were acquired from the Health and Aging Brain Study: Health Disparities (HABS‐HD). MBI‐affective dysregulation (MBI‐affect) was operationalized based on a diagnosis of depression and/or anxiety within 5 years of baseline. Comparator groups included participants with no previous diagnosis of depression or anxiety (No‐affect) and those with a diagnosis >5 years prior to baseline (Non‐MBI‐affect). Plasma concentrations of inflammatory biomarkers IL5, IL6, IL10, and TNFα were obtained using single molecule array (SiMOA) technology. Linear regressions modeled the cross‐sectional association between NPS group (predictor) and inflammatory biomarker level (outcome), adjusted for age, sex, education, race, and cognitive diagnosis (normal cognition or mild cognitive impairment (MCI)).

**Result:**

The sample comprised 74 MBI‐affect (age = 62.4±7.5; 81.1% female; 23.0% MCI), 143 Non‐MBI‐affect (age = 64.3±7.1; 76.2% female; 21.0% MCI), and 396 No‐affect participants (age = 66.7±8.9; 51.8% female; 13.6% MCI). Those with MBI‐affect had significantly higher baseline levels of plasma IL6 (b = 3.10, 95%CI:1.01‐5.19, *p* = 0.004) and TNFα (b = 1.66, 95%CI:0.43‐2.89, *p* = 0.008) compared to No‐affect, after multivariable adjustment. Biomarker levels in Non‐MBI‐affect did not differ significantly from the No‐affect group. For IL5, while MBI‐affect participants did not significantly differ from No‐affect (b = 0.03, 95%CI:‐0.002‐0.07, *p* = 0.06), the magnitude and direction of the effect were similar to the other biomarkers (Table 1).

**Conclusion:**

Compared to No‐affect, MBI affective dysregulation was associated with higher levels of inflammatory biomarkers IL6 and TNFα, while Non‐MBI‐affect showed no association. The link between MBI affective dysregulation and neuroinflammation further informs our understanding of the biological underpinnings of MBI. These findings add to the evidence base for MBI as a clinically relevant approach to identify high‐risk individuals for cognitive decline and incident dementia.